# Gene-coexpression network analysis identifies specific modules and hub genes related to cold stress in rice

**DOI:** 10.1186/s12864-022-08438-3

**Published:** 2022-04-01

**Authors:** Zhichi Zeng, Sichen Zhang, Wenyan Li, Baoshan Chen, Wenlan Li

**Affiliations:** 1 State Key Laboratory for Conservation and Utilization of Subtropical Agro-Bioresources, Guangxi University, Nanning, China; 2 College of Life Science and Technology, Guangxi University, Nanning, China; 3 College of Agriculture, Guangxi University, Nanning, China

**Keywords:** Cold stress, Transcriptome, WGCNA, Hub genes

## Abstract

**Background:**

When plants are subjected to cold stress, they undergo a series of molecular and physiological changes to protect themselves from injury. Indica cultivars can usually withstand only mild cold stress in a relatively short period. Hormone-mediated defence response plays an important role in cold stress. Weighted gene co-expression network analysis (WGCNA) is a very useful tool for studying the correlation between genes, identifying modules with high phenotype correlation, and identifying Hub genes in different modules. Many studies have elucidated the molecular mechanisms of cold tolerance in different plants, but little information about the recovery process after cold stress is available.

**Results:**

To understand the molecular mechanism of cold tolerance in rice, we performed comprehensive transcriptome analyses during cold treatment and recovery stage in two cultivars of near-isogenic lines (9311 and DC907). Twelve transcriptomes in two rice cultivars were determined. A total of 2509 new genes were predicted by fragment splicing and assembly, and 7506 differentially expressed genes were identified by pairwise comparison. A total of 26 modules were obtained by expression-network analysis, 12 of which were highly correlated with cold stress or recovery treatment. We further identified candidate Hub genes associated with specific modules and analysed their regulatory relationships based on coexpression data. Results showed that various plant-hormone regulatory genes acted together to protect plants from physiological damage under short-term low-temperature stress. We speculated that this may be common in rice. Under long-term cold stress, rice improved the tolerance to low-temperature stress by promoting autophagy, sugar synthesis, and metabolism.

**Conclusion:**

Through WGCNA analysis at the transcriptome level, we provided a potential regulatory mechanism for the cold stress and recovery of rice cultivars and identified candidate central genes. Our findings provided an important reference for the future cultivation of rice strains with good tolerance.

**Supplementary Information:**

The online version contains supplementary material available at 10.1186/s12864-022-08438-3.

## Background

Rice is one of the most important food crops and has a very wide planting area all over the world. It feeds most of the global population [1]. As a model plant, it plays an important role in research on genetic breeding and agricultural ecosystems. Rice-genome sequencing was completed for the first time in 2002, thereby providing abundant resources for studies on rice functional genomics [2, 3]. According to phylogenetic studies, rice can be divided into japonica and indica [4]. Indica is more sensitive to low temperature than japonica [5, 6].

Abiotic stresses such as salt stress, flooding, drought, freezing, and heavy-metal ions exert adverse effects on plant growth and reproduction [7–11]. Amongst them, cold stress primarily inflicts damage to plants during seed germination and morphogenesis [12–14]. After a long period of cold acclimation, plants in temperate regions are tolerant to cold stress, whereas plants adapted to tropical and subtropical environments are generally more sensitive to low temperature [15]. Low temperature seriously affects the geographical distribution of plants and reduces agricultural productivity [16].

At low temperature, the activities of starch hydrolysis and sugar metabolism are weakened, and the germination rate of rice seeds decreases [17]. Rice suffers from cold injury at the seedling stage, causing leaves to be discoloured, yellowed, curled, and wilted, as well as the growth to slow down and the biological cycle to be prolonged [18]. In the reproductive stage, low temperature affects microspore development, resulting in reduced pollen grains during anther development and improved spikelet sterility, thereby seriously reducing the rice yield [19]. Therefore, cultivating rice with excellent cold resistance is an important goal of rice breeding.

Under low-temperature stress, the chlorophyll content, plastid damage, and cell membrane fluidity of cold-sensitive plants decrease, whereas the unsaturated fatty acid synthesis, reducing sugar content, and cell metabolism of cold-tolerant plants increase [20]. RNA-sequence (RNA-seq) technology is a powerful tool for understanding the response mechanism of rice to cold stress. Many cold-responsive genes have been screened from rice, *Arabidopsis thaliana*, wheat, and sorghum by using structural gene-expression maps, and different regulation pathways in response to cold stress have been analysed. Plant cold-stress response is a complex biological process controlled by multiple genes. High-throughput transcriptome sequencing can provide new insights into the pathways and interaction networks of plant cold stress at the transcriptome level [21]. Amongst them, RNA-seq technology is widely used in cold-resistance gene screening and cold-resistance response regulation amongst different plants [22–26]. Cold stress can induce the expression of AP2-domain protein CBF family of transcription factors (CBFs), thereby activating the expression of downstream cold-responsive genes [27]. Inducer of CBF expression 1 is an upstream transcription factor that regulates CBF gene expression under cold stress [28]. Cold-stress response gene-expression pathways are classified as ABA dependent or ABA independent. ABF/AREB-dependent gene-expression pathways are ABA dependent, DREB1/CBF-dependent cold-response gene-expression pathways are ABA independent, and the two pathways are cross-linked and interdependent [29].

The genetic factors controlling this trait can be well revealed using near-isogenic rice varieties with similar genetic backgrounds and significant phenotypic differences of some traits [30]. In our early studies, we have identified some cold-tolerance (quantitative trait locus) QTLs, several of which have been successfully mapped using cold-tolerant QTL substitution line population mapping [31, 32]. We have sequenced the whole genome of cold-tolerant diploid wild rice DP30 and parents 9311 and DC907 in our laboratory. The whole-genome sequence alignment of the three sequences shows that cold-tolerant wild rice has obvious specific sequence segments. A near-isogenic cold-tolerant line is obtained by introducing the cold-tolerance QTL into 9311, which provides a suitable material to study the molecular physiological mechanism of cold-tolerance gene and a reliable material basis for the cold-tolerance breeding and utilisation of rice.

In the present study, we analysed the transcriptome of 9311 and DC907 during cold treatment and recovery. Transcriptome-sequencing technique was used to analyse the transcriptomes of two rice cultivars treated at different time intervals. These data provided very comprehensive and systematic information on transcriptome changes in different rice varieties under moderate and higher levels of low-temperature stress, as well as transcriptome changes during recovery after different degrees of low-temperature stress. Using these data, we performed weighted gene-coexpression network analysis (WGCNA) analysis to screen out modules that were highly correlated with different treatments, as well as coexpressed genes and candidate central genes in the modules. Our findings provided new insights into the molecular mechanism of cold stress and post-frostbite recovery in rice.

## Results

**Fig. 1:**
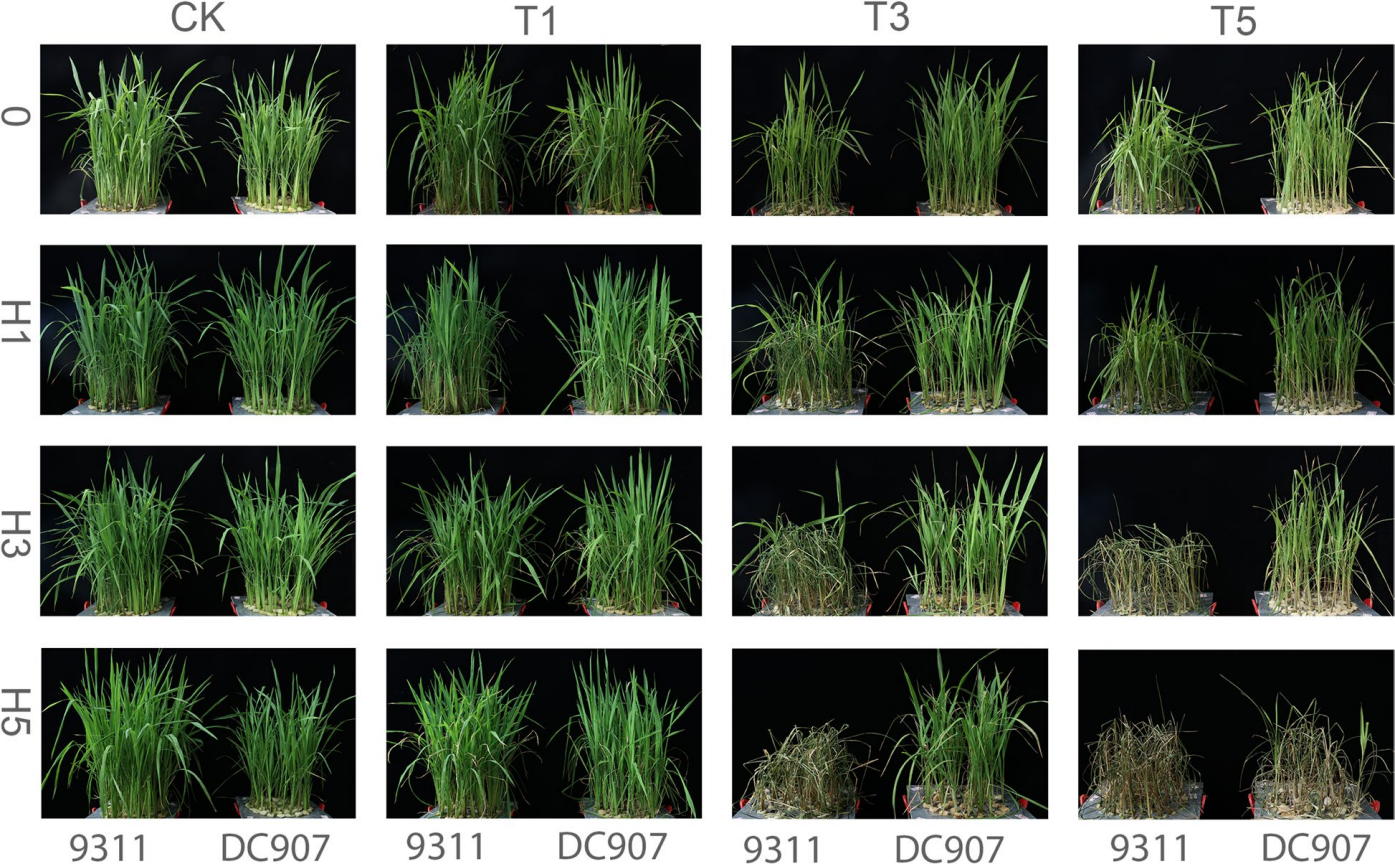
CK is the control group, T1 stands for 1 day of cold-stress treatment at 8 ℃, T3 stands for 3 days of cold-stress treatment at 8 ℃, T5 stands for 5 days of cold-stress treatment at 8 ℃, 0 stands for only cold-stress experiment, H1 stands for one day of normal temperature recovery after cold stress, H3 stands for 3 days of normal temperature recovery after cold stress, and H5 stands for 5 days of normal temperature recovery after cold stress

The phenotypic changes of rice seedlings at different stress stages are shown in Fig. 1. After 1 day of cold treatment, the appearance of 9311 and DC907 did not change significantly, and their growth conditions were similar, indicating that 9311 and DC907 had the ability to withstand short-term low temperatures. After 3 days of cold treatment, the leaves of 9311 curled and wilted, whereas those of DC907 did not. After 5 days of cold treatment, the leaves of 9311 began to wilt and turn yellow, whereas those of DC907 had a lower wilting degree. These results showed that DC907 had better low-temperature resistance than 9311 under longer low-temperature conditions. In the process of recovery test, with prolonged recovery time, CK and T1 treatment grew normally. In the recovery process of T3 treatments, 9311 leaves gradually turned yellow with prolonged recovery time, and all plants died at the end of 5 days of recovery. Conversely, for DC907, most original curled leaves gradually recovered and only a few leaves finally turned yellow and died with prolonged recovery time. In the recovery process of T5 treatment, leaf curl appeared in most rice varieties. With prolonged recovery time, all 9311 plants gradually turned yellow and eventually died at 5 days of recovery, whereas DC907 also turned yellow and died at the same time. After 5 days of recovery, only a few plants could maintain normal growth. Moreover, the cold resistance of DC907 was stronger than that of 9311, and the recovery ability was stronger after cold injury.

### Global transcriptome analysis of rice varieties

To avoid increasing the difficulty of data analysis and the analysis error caused by comparing multiple similar groups, some treatment groups were selected for transcriptome sequencing and subsequent analysis based on the above phenotypic observations. The materials and methods for transcriptome sequencing are described in detail. The transcriptomes of 10 experimental treatments (AT1, AT1H1, AT3, AT3H1, BT1, BT1H1, BT1H3, BT3, BT3H1, and BT3H3) (9311 and DC907 to represent A and B) and 2 blank controls (ACK and BCK) were sequenced.

RNA-seq analysis was performed using rice aboveground-tissue samples. Each treatment contained three biological replicates of 36 samples. The number of raw reads ranged from 39.72 million to 47.26 million, and the number of clean reads obtained by data filtering ranged from 38.96 million to 46.05 million. The percentage of Q30 base to total base ranged from 91.59% to 95.24% (Additional file 1). Rice reference genomes were compared using HISAT2 software [33], and results showed that the relative efficiency of 9311 and DC907 was 82.96%–95.15% and 89.89%–95.15% respectively. Amongst them, the relative efficiency of AT1_3 with genome was low (67.51%) (Additional file 2). However, we consider it as sequencing based on the previous quality-control results and sample correlation analysis. Considering the lack of depth, we did not believe it would adversely affect the follow-up analysis and decided to use it for such analysis.

**Fig. 2:**
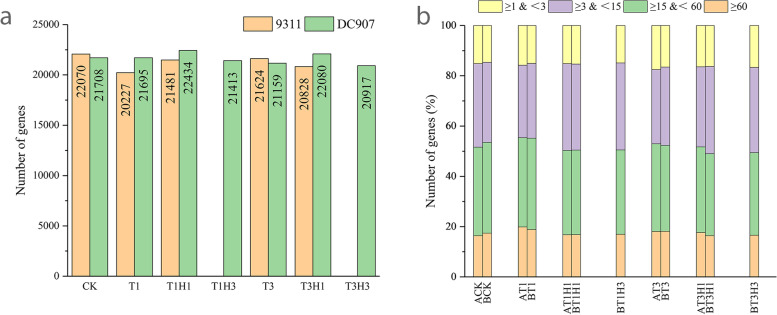
Gene expression of 9311 and DC907 at different treatment stages. Total number of genes expressed (**a**) and fraction of genes expressed at different expression levels (based on FPKM) (**b**) under different treatment in 9311 and DC907 are shown in the bar graphs

A total of 43 255 genes were identified by Cufflinks and Cuffcompare, including 40 745 annotated genes and 2509 new genes. Overall, about 51.6% of the genes were expressed in at least one of the 12 samples (fragments per kilobase of transcript per million [FPKM] > 1). The number of expressed genes ranged from 46.7% (T1) to 51.0% (CK) in 9311 and from 48.4% (T3H3) to 51.9% (T1H1) in DC907 (Fig. 2a). In all treatments, the number of genes with low (1 < FPKM < 3), medium (3 < FPKM < 15), high (15 < FPKM < 60), and very high expression (FPKM < 60) were similar (Fig. 2b). The number of genes with low expression (CK and T1) had the highest gene expression in the two cultivars.

### Identification of differentially expressed genes (DEGs) in different treatments

To investigate the transcriptional differences between two rice varieties at different treatment stages, we identified the DEGs of the two rice varieties at each treatment stage. Each treatment stage was compared with CK, and DEGs were filtered with padj < 0.5. Based on this formula, 4178 and 6054 of genes were significantly differentially expressed in 9311 and DC907. Amongst 9311 cold-sensitive rice cultivars, 2911 DEGs were identified in AT1, with the largest number of DEGs; 174 DEGs were identified in AT1H1, with the least number of DEGs. In the cold-insensitive rice cultivar DC907, 4380 DEGs were identified in BT3, with the largest number of DEGs; 285 DEGs were identified in BT1H3, with the least number of DEGs.

Interestingly, the proportion of upregulated genes in AT1 was 70% and that of upregulated genes in AT3 was 51%. The proportion of upregulated genes in all differential genes decreased with increased cold treatment time; the proportion of upregulated genes in BT1 was 74%, and that of upregulated genes in BT3 was 60%. The proportion of upregulated genes in DC907 decreased with increased cold treatment time. The proportion of upregulated genes in DC907 was slightly higher than 9311 on the first day of cold treatment, but the difference enlarged on the third day of cold treatment. The changes in number of these abnormal genes indicated that they may have had different molecular stress mechanisms at different stages of cold treatment. We also found that the number of DEGs in recovery treatment after 3 days of cold treatment was higher than that in recovery treatment after 1 day of cold treatment (Table 1). Results showed that with increased effect of cold treatment on rice, more genes were needed to participate in the recovery process to restore normal rice growth.

**Table 1:** Numbers of DEGs in each experimental-processing stage

Sample	Up	Down	Total
AT1 vs ACK	2034	877	2911
AT1H1 vs ACK	143	31	174
AT3 vs ACK	576	528	1104
AT3H1 vs ACK	522	501	1023
BT1 vs BCK	1612	558	2170
BT1H1 vs BCK	73	159	232
BT1H3 vs BCK	182	103	285
BT3 vs BCK	2658	1722	4380
BT3H1 vs BCK	292	300	592
BT3H3 vs BCK	345	608	953

### Functional-enrichment analysis of DEGs

**Fig. 3:**
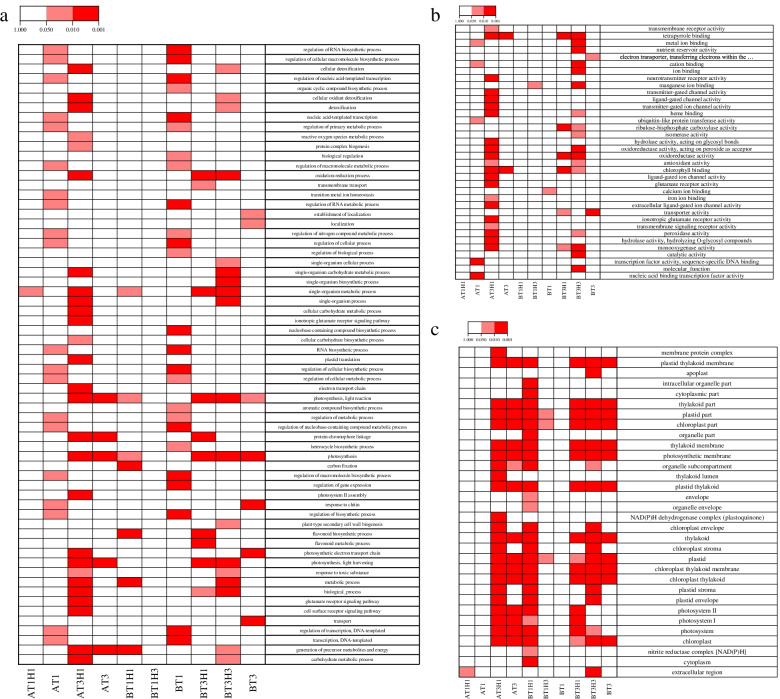
Enriched Gene Ontology (GO) terms of DEGs in 9311 and DC907 cultivars: biological process (**a**), molecular function (**b**), and cellular component (**c**). The colour scale at the top represents significance (corrected *P* value)

A total of 7 506 DEGs were screened out after subcooling stress and recovery experiments. To understand the regulatory effects of these genes in different experimental approaches, Gene Ontology (GO) enrichment analysis was performed on DEGs in different experimental groups. At the T1 stage, DEGs were categorised in different GO terms, including RNA biosynthetic process, cellular metabolic process, and primary metabolic process. Results indicated that the transcriptional, translational, and metabolic activities of rice increased significantly within 1 day of cold treatment. At the T3 stage, the GO terms related to photosynthesis were significantly enriched. This finding showed that after a long period of cold treatment, the photosynthesis system of plants was damaged. In the process of recovery, the significantly enriched GO terms showed difference between the two rice varieties. In 9311 cultivars, many GO terms related to antioxidant activity were significantly enriched at the T1H1 and T3H1 stages, and it was found that a large number of genes involved in antioxidant activity in T3H1 were depressed. In DC907 cultivars, a significant difference existed in GO enrichment between recovery 1 and 3 days. At the T1H1 and T3H1 stages, many specific genes were enriched in flavonoid metabolic process and flavonoid biosynthetic process GO terms. At the T3H1 stage, GO terms related to ROS scavenging were enriched, such as detoxification, response to toxic substance, and oxidation–reduction process (Fig. 3). At different treatment stages, rice may resist low-temperature stress through various regulatory methods.

### Identification of DEGs at different treatment stages of rice seedlings

**Fig. 4:**
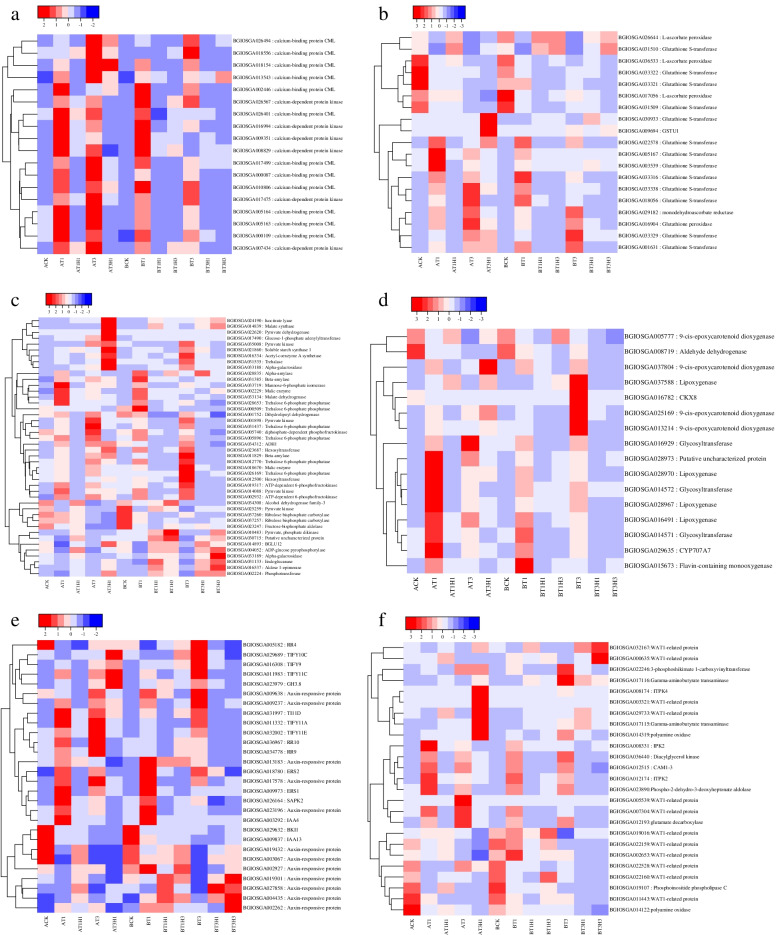
Differential expression of members of selected gene families in rice cultivars: calcium signal transduction (**a**), biosynthesis of antioxidants (**b**), sugar synthesis and metabolism (**c**), plant-hormone biosynthesis (**d**), plant-hormone signal transduction (**e**), and some other functional genes (**f**). The colour scale on the top represents Z-score

We analysed the expression profiles of genes related to plant-hormone biosynthesis, plant-hormone signal transduction, ROS scavenging, and plant antifreeze biosynthesis. CDPKs are a class of protein kinases that exist only in plants and some protozoa. Calcium participates in the signal-transduction process of calcium ion as a second messenger [34] and plays an important role in regulating plant growth and development signals and stress signals [35, 36]. Six CDPK-coding genes were upregulated during cold stress (Fig. 4a). Notably, the overall expression of CDPK-coding genes in 1 day of cold treatment was higher than that in 3 days of cold treatment. CML family proteins containing predicted Ca^2+^-binding EF-hand motif are potential calcium sensors [37]. Under abiotic stresses such as high temperature, low temperature, hypoxia, and darkness, the expression of CML protein significantly increases [38]. A total of 12 CML coding genes were identified to be upregulated under cold stress. The total expression level in 9311 was higher than that in DC907. Calcium participates in the signal-transduction pathway of rice cold-stress response, which is widespread in sensitive and cold-insensitive rice lines. Calcium signalling pathway primarily plays a regulatory role in the early cold-stress process of rice.

Reactive oxygen species (ROS) are important intermediates in the plant abiotic-stress process. An appropriate amount of ROS can regulate abiotic-stress responses in plants through signal-transduction pathways. With prolonged stress time, ROS accumulation inflicts oxidative damage to plants. During the cold treatment and restoration of rice, numerous differentially expressed antioxidant synthesis genes were screened (Fig. 4b). Glutathione transferase is ubiquitous in plants. It plays an important role in plant stress response by preventing the oxidative damage of ROS [39, 40]. Six glutathione S-transferase coding genes were overexpressed in AT1. At the AT3 stage, three glutathione S-transferases, one glutathione peroxidase, and one monodehydroascorbate reductase increased after cold treatment. Five glutathione S-transferase coding genes were induced in AT1. Two glutathione S-transferase coding genes BGIOSGA033329 and BGIOSGA001631, one glutathione peroxidase coding gene BGIOSGA016904, and one single dehydroascorbate reductase coding gene BGIOSGA029182 were induced to be expressed in BT3. In *Arabidopsis*, regulating the redox state through the ascorbic acid–glutathione cycle can reduce oxidative stress under many abiotic stresses [41]. Results showed that the ASA-GSH cycle played a positive role in the regulation of cold stress in rice, and with prolonged cold-treatment time, more enzymes became involved in the induction of expression.

Sugar is an important regulator of plant growth, morphogenesis, and expression. It also plays an important role in maintaining basic plant-life activities and signal transduction. Trehalose accumulation in plants can improve plant tolerance to abiotic stress [42, 43]. In the present study, many genes involved in trehalose biosynthesis were induced and expressed. Two trehalose 6-phosphate phosphatase encoding trehalose 6-phosphate were significantly induced to be expressed in cold-treated day (AT1 and BT1) (Fig. 4c). It can remove phosphate from trehalose phosphate to produce free trehalose [42]. The expression of Trehalase gene BGIOSGA031535 increased in AT3H1 and BT3. Its main biological function is to hydrolyse trehalose into glucose. Four trehalose 6-phosphate phosphorylase coding genes were identified in BT3, which were upregulated and differed from the above genes. These results indicated that trehalose played an important role in maintaining the normal life activities of rice. Under cold stress, different genes were expressed at different treatment stages, causing rice to have cold tolerance. The role of amylase in plants is to hydrolyse starch and ultimately decompose it into glucose. The expression of one α-amylase and two β-amylases were found to be induced during treatment. BGIOSGA028835 and BGIOSGA031385 were induced to be expressed during the first day of cold treatment, and BGIOSGA011829 was induced to be expressed in BT3. It can mediate maltose accumulation under freezing stress, thereby helping to protect the photosynthetic electron-transport chain [44, 45]. Two genes encoding ribulose bisphosphate carboxylase (ribulose bisphosphate carboxylase) inhibited during treatment were involved in the immobilisation of carbon dioxide and the oxidation of pentose substrates in plants. Results showed that the photosynthesis capacity of plants decreased during cold treatment and short-term recovery, consistent with previous experimental observations and functional-enrichment analysis.

We found that most plant-hormone biosynthesis and signal-transduction genes were upregulated during cold stress, suggesting that cold-stress response was the main process of cold tolerance in rice, and that the regulation of gene expression may play only an auxiliary role during recovery. Four NCED coding genes were significantly differentially expressed; amongst them, NCED was the rate-limiting enzyme in the process of ABA biosynthesis [46]. BGIOSGA025169 and BGIOSGA013244 were upregulated in BT3, whereas BGIOSGA083804 was induced during DC907 cold stress and in 9311 for 3 days (Fig. 4d). This finding indicated that the ABA biosynthesis pathway played an important role in the cold tolerance of rice. CYP707A7 participated in the oxidative degradation of abscisic acid. BGIOSGA029635, the coding gene of CYP707A7, was significantly induced during cold treatment. The expression of CYP707A7 in AT1 was higher than that in BT1H1 and AT1H1. We speculated that rice needed to accumulate more ABA to participate in cold signal transduction during 3 days of cold treatment. SAPK2 plays an important role in ABA signal transduction. We found that the SAPK2 coding gene BGIOSGA026164 was induced to be expressed in two cultivars during cold treatment, and that the expression level was higher than 3 days in 1 day (Fig. 4e). In rice, SAPK2 was strongly induced under salt and drought stress, and SAPK2 gene overexpression could reduce rice sensitivity to salt [47]. Jasmonic acids (JAs) are an important component of plant hormones. Lipoxygenase is a key enzyme in the biosynthesis of JA. In the current work, we found that four genes encoding lipoxygenase were induced to be expressed during cold stress. BGIOSGA037588 was highly expressed in BT3 and the other three in AT1. TIFY protein is a repressor of JA reaction [48]. Six TIFY coding genes were identified to be differentially expressed during the treatment. Notably, TIFY protein was very highly expressed in BT3 compared with the DC907 treatment group. In the 9311 treatment group, they were expressed in AT1, AT3, and AT3H1 to varying degrees. Cytokinin dehydrogenase 8 (CKX8) can catalyse cytokinin oxidation in plants. BGIOSGA016782, the coding gene of CKX8, was upregulated in BT3, and its expression was higher than that of other treatments. Flavin-containing monooxygenase may be involved in auxin biosynthesis in rice. We found that BGIOSGA015673, the coding gene of flavin-containing monooxygenase, was significantly induced to be expressed during cold treatment for 1 day. In this study, we also found that nine genes encoding auxin-response protein(ARF) were significantly differentially expressed during treatment. IAA4 was upregulated during the first day of cold stress, IAA3 was inhibited in each treatment, and other ARF genes were inhibited or induced to varying degrees during the recovery treatment of DC907. Results showed that auxin signal transduction played an important role in cold stress and recovery. Two genes encoding the ethylene-response sensor proteins ERS1 and ERS2 were induced to be expressed during cold treatment. BGIOSGA009308 and BGIOSGA009973 were significantly upregulated during cold treatment. Through the above analysis of plant-hormone biosynthesis and signal-transduction pathways, we found complex synergistic regulatory networks and signal-transduction pathways amongst ABA, JA, auxin, and ethylene, which enabled rice to withstand low temperatures.

In addition to the aforementioned pathways that had been extensively studied under abiotic stress, we screened several genes that were differentially expressed during cold treatment and recovery, and these genes may play important roles in regulating cold tolerance or recovery in rice. From literature, we can find that Phosphoinositide phospholipase C coding gene is significantly downregulated during cold treatment. It can mediate the production of the second messenger molecule diacylglycerol and inositol 1,4,5-triphosphate through the phosphatidylinositol signalling pathway [49]. Herein, we also found that one gene encoding Inositol polyphosphate multikinase (IPK2) and one gene encoding Inositol-tetrakisphosphate 1-kinase 2 (ITPK2) were upregulated during cold treatment. Notably, their expression level on 1 day after cold treatment was higher than that 3 days after cold treatment and BT3. Both genes were involved in inositol phosphate metabolism [50, 51]. CAM1-3 was induced to be expressed during cold treatment, and the highest expression was in AT1. The differential expression of these genes suggested that the activation of the phosphoinositol signalling pathway may play a potential role in maintaining cell homeostasis under cold stress. Polyamines are plant growth regulators that participate in important physiological processes, such as plant growth and development, sex differentiation, aging, and environmental adaptation [52, 53]. We found that two polyamine oxidase coding genes were significantly differentially expressed during treatment. BGIOSGA014319 was induced in BT3 and BGIOSGA014122 was significantly downregulated during the recovery of 9311 and DC907 after 3 days of cold treatment (Fig. 4f). Polyamine oxidase participates in secondary amino oxidation and plays an important role in regulating intracellular polyamine solubility [54]. After cold treatment, we found that the genes involved in spermidine biosynthesis showed an induced expression trend. Spermine may play an antifreezing role in rice. Gamma-aminobutyric acid (GABA) is a non-protein amino acid converted from l-glutamic acid. Aluminum activates malate transporter activity to regulate plant growth, development, and stress-response signals [55–57]. The expression of the glutamate decarboxylase gene BGIOSGA012193 was significantly upregulated after cold treatment and increased with prolonged cold-treatment time. Glutamic acid decarboxylase can catalyse the conversion of l-glutamic acid to GABA. According to the above experimental results, GABA and spermidine may play the role of antifreeze protector in rice and induce expression in cold treatment.

**Fig. 5:**
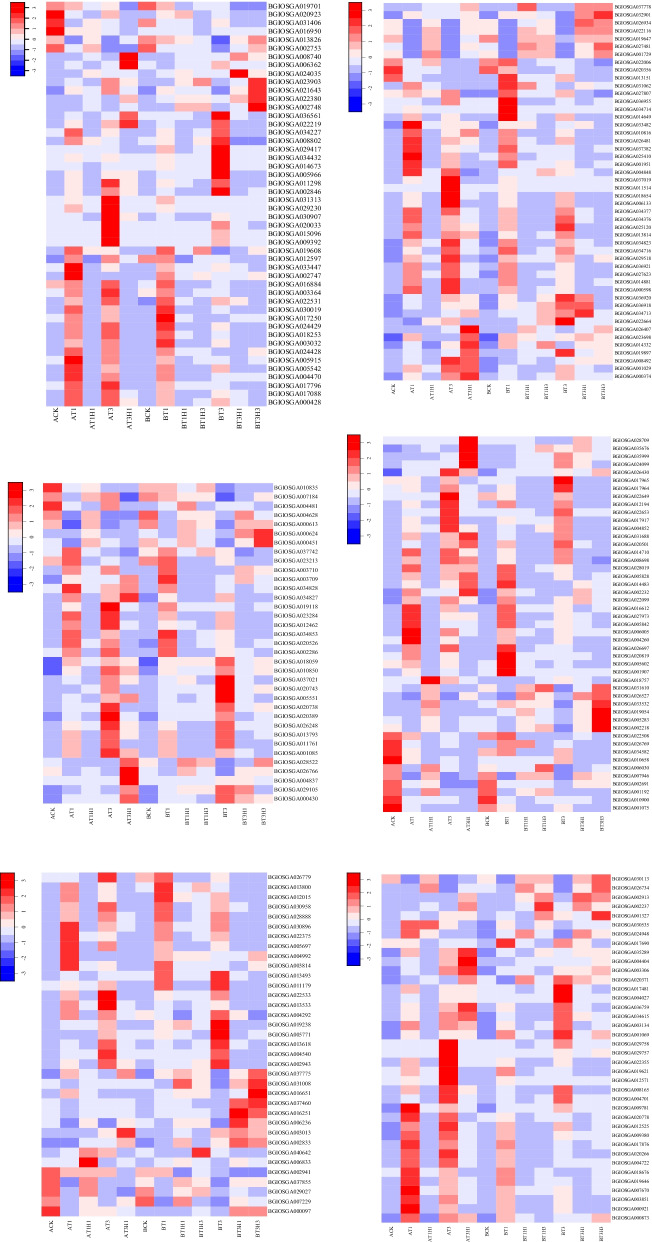
Heatmaps showing the expression profiles of TF family members selected from two rice varieties during cold-stress treatment and recovery

Numerous transcription factors are reportedly involved in abiotic stress [58–60]. Two transcription-factor encoding genes, namely, the dehydration-responsive element binding factors DREB1B and DREB1C, were significantly upregulated at the BT3 stage. Interestingly, DREB1B was also significantly induced in 9311 cultivars during 1 day of cold treatment (Fig. 5 AP2/EREBP). The high expression of DREB1B may endow tobacco plants with resistance to high salt, cold, and drought stress, and inserting *OsDREB1B* gene into tobacco can make plants more tolerant to oxidative and freezing stress than the wild type [61]. In *Arabidopsis*, DREB1C negatively regulates the expression of CBF1/DREB1B and CBF3/DREB1A and plays a negative role in stress tolerance [62]. However, in rice cultivars, DREB1C may play an essential role in cold tolerance. In DC907 cultivars, stress-induced transcription factor NAC1 is induced after cold stress (Fig. 5 NAC). It is also upregulated in AT1. In rice, *OsPP18* is regulated by SNAC1 and regulates ROS homeostasis through the abscisic acid-independent pathway, thereby conferring oxygen stress and drought tolerance [63]. Ten putative WRKY transcription factors were filtered from all upregulated DEGs (Fig. 5 WRKY). WRKYs had different biological functions in plants and participate in response to biotic and abiotic stresses [64–66].

### Analysis of gene-coexpression network

**Fig. 6:**
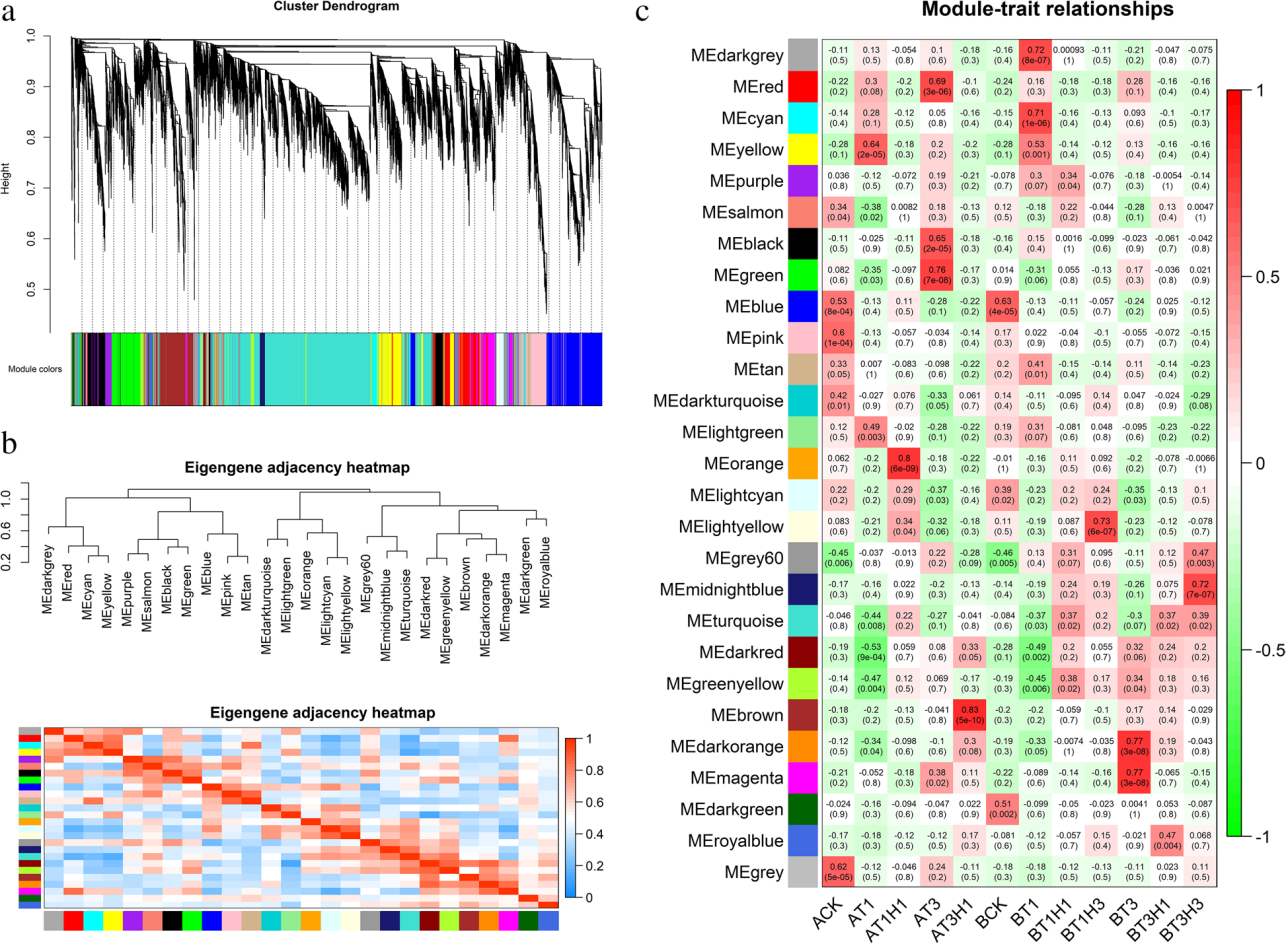
Coexpression network of different treatments in rice cultivars. (**a**) Hierarchical cluster tree showing coexpression modules identified by WGCNA. Each leaf in the tree represents one gene. The major tree branches constitute 36 modules, labeled with different colours. (**b**) Hierarchical clustering of module genes that summarize the modules found in the clustering analysis. Branches of the dendrogram group together with genes that were positively correlated. (**c**) Each row corresponds to a module eigengene (correlation between a column and a trait). Each cell contains the corresponding correlation and P value. The table was colour coded by correlation in accordance with the figure

We used WGCNA analysis to further understand the gene expression of the two near-isogenic lines rice in different treatments and to screen out the characteristic genes related to cold tolerance. After filtering raw data in materials and methods, 19 894 genes were retained for WGCNA analysis. The coexpression network was constructed based on the gene expression of all samples. The module was a cluster of highly interconnected genes, agreeing that the genes in the cluster were highly correlated with one another. A total of 26 modules were identified for different colours, and the grey for genes meant no assignment to any module. Considering the specific expression of genes in different samples, the correlation coefficients amongst different samples of the 26 modules differed. Notably, 13 out of the 26 coexpression modules showed high correlation with single sample (r > 0.6). WGCNA analysis can also be used to construct gene-expression networks, where each node represents a gene and the line (edges) represents the correlation between two connecting genes (Fig. 6). Hub genes refer to genes with high connectivity in modules. Hub genes with high connectivity are usually regulatory factors (the upstream position of the regulatory network), whereas those with low connectivity are usually downstream genes in the regulatory network (e.g., transporters, catalytic enzymes, etc.). Hub genes are linked to more genes in the interaction network and have a high KME value.

In the yellow module, 1410 genes highly correlated with AT1 were identified. A total of 39 specific genes of AT1H1 were found in the orange module. A total of 1097, 1095, and 1114 genes were found to be highly correlated with AT3 in red, black, and green. A total of 1455 AT3H1-specific genes were identified in the brown module. In the dark-grey and cyan modules, 62 and 210 genes were found to be highly correlated with AT1. A total of 114 specific genes of BT1H3 were identified in the light-yellow module. In the dark-orange and magenta modules, 35 and 888 genes highly associated with BT3 were found. In the midnight-blue module, 206 genes were found to be highly correlated with BT3H3.

In the yellow module, 80 gene-coding transcription factors were identified, including 5 bHLH transcription factors, 3 bZIP transcription factors, 4 HOX transcription factors, 9 AP2-EREBP transcription factors, 2 MADS transcription factors, 5 MYB transcription factors, 8 NAC transcription factors, 1 TIFY protein, and 6 WRKY transcription factors. NAC family transcription factors (BGIOSGA001951) were identified as candidate Hub genes for this module. In the cyan module, 13 presumptive transcription-factor genes were identified, including 1 ARF, 1 bZIP, 1 bHLH, 1 NAC, and 2 Tubby-like F-box domain transcription factors. Calcium-transporting ATPase and auxin-response protein were identified as candidate central genes for this module.

As shown in the figure, the dark-orange and magenta modules had relatively high correlation, and both of them were presumably BT3-specific genes. In the dark-orange module, a gene encoding C3H transfer factor (BGIOSGA004750) was identified. Peptidylprolyl isomerase was identified as a candidate central gene for this module. In the magenta module, four transcription factors of AP2 domain, two bHLH, two bZIP, one MYB, two NACs, and three WRKY transcription factors were found in the magenta module. BGIOSGA002846 and membrane proteins associated with secretory vectors were identified as candidate central genes for this module.

Midnight-blue and turquoise modules were highly correlated, and both modules were positively correlated with BT3H3, so the genes in both modules may be BT3H3-specific genes. In the midnight-blue module, two presumptive transcription factors, one AP2 family transcription factor, and one bHLH transcription factor were found. BGIOSGA015677 is an ubiquitin-transferase coding gene identified as a candidate central gene for this module. In the turquoise module, 63 transcription factors were identified, including 5 bHLH, 5 bZIP, 9 MYB, 3 CH3, 5 HB, 9 NAC, and 4 WRKY transcription factors. Before screening, Hub genes were not annotated, and BGIOSGA021307 and BGIOSGA021806 genes were relatively high in expression. We speculated that these two genes were candidate central genes of the turquoise module.

In the cluster diagram, black and green are the specific module genes of AT3. In the black module, we identified 26 presumptive transcription-factor proteins, including 2 bHLH, 3 bZIP, 2 MYB, 5 NAC, and 3 WRKY transcription factors. A LOB transcription-factor coding gene (BGIOSGA010454) and phosphatidyl cytidyltransferase were identified as candidate central genes for this module. In the green module, 60S ribosomal proteins l44, L18a, L11, and SPT4 homologues were identified as candidate central genes for this module. Brown was an AT3H1 characteristic module gene. In this module, we identified 43 presumed transcription-factor proteins, including three AP2 domain transcription factors, one bHLH, five bZIP, two HBs, seven MYB, four NAC, three TIFY proteins, six WRKY, and one leucine zipper domain homologue protein. Aspartate aminotransferase, Peroxygenase, and Reticulon-like protein were identified as candidate central genes for this module.

Dark-grey was a characteristic module gene of BT1. In this module, we identified four presumed transcription-factor proteins, including one C2H2, one G2-like, one AP2 domain protein, and one HB transcription factor. DelLA protein SLR1 and xylan transglucanase/hydrolase were identified as candidate central genes for this module. Light yellow was a characteristic module gene of BT1H3, in which two predictive coding transcription-factor genes zf-HD and bHLH were identified. ITPK3 was identified as a candidate central gene for this module.

### Validation of the expression of selected genes

In 9311 and DC07, we selected 14 genes for qRT-PCR verification (Figure S1), and the primer information is shown in Table S1. The results suggested high-reliability of the RNA-seq data.

#### Discussion

The molecular mechanism of rice's dynamic change from cold stress to recovery is unclear. RNA-seq technology was used to analyse the transcriptome data of two rice varieties at different processing stages, and the relationship between low-temperature stress and the survival of rice during the recovery period was studied. By studying the molecular mechanism of rice recovery after different degrees of cold stress, we can improve our understanding of rice-regulation mechanism under abiotic stress and provide theoretical basis for the subsequent cultivation of tolerant varieties. At least 80% of genes the total samples were expressed based on the rice reference genome. These transcriptome data can describe the life activities of rice in different experimental treatments as a whole. Combining WGCNA with traditional transcriptome analysis, we identified some core regulatory elements related to cold tolerance and post-frostbite recovery of rice, as well as specific modules in different treatments.

Rice varieties 9311 and DC907 had slightly curled leaves at T1 stage and grew normally at the subsequent recovery stages of T1H1 and T1H3. We speculated that rice may have similar stress-regulation mechanisms to protect rice from damage under short-term cold stress. With prolonged cold-stress time at the T3 stage, rice leaves showed obvious curling and 9311 gradually died at the T3H1 and T3H3 recovery stages, whereas DC907 only partially died with prolonged cold-stress time. Phenotypic differences between 9311 and DC907 at the recovery stage may be the result of DEGs at the T3 and T3H1 stages.

#### Molecular mechanism of cold stress in two rice cultivars under short-term cold treatment

Rice cultivars 9311 and DC907 had the same phenotypic changes during 1 day of cold treatment, and all rice seedlings survived and could grow and reproduce normally during recovery after one day of cold treatment. Short-term low-temperature stress could be resisted by 9311 and DC907. We speculated a similar mechanism of cold response during this period, and it continued to play a role during recovery after one day of stress. The survival rate of DC907 was higher than 9311 in the subsequent recovery process. We speculated that the difference in gene expression at the T3H1 and T3H3 stages may be primarily due to the differential gene expression of 9311 and DC907 at the T3 stage, and a small part was determined by the differential gene expression in the self-treatment stage.

Ca^2+^ signal-transduction genes including four CDPK and seven CML coding genes were upregulated at T1. CDPK reportedly regulates plant growth and development, as well as biotic and abiotic stresses. The overexpression of *OsCDPK7* gene in rice can increase salt and drought tolerance [67]. Some researchers have analysed the CML family proteins in *A. thaliana* and found that CML24 can be expressed in different organs and respond to various stimuli. Ca^2+^ sensor may also respond to ABA and salt stress [38]. The high expression of genes related to calcium signal transduction at T1 indicated that cold-sensitive 9311 and cold-insensitive rice DC907 could respond to cold signals in a short period of cold-stress treatment. Genes involved in the phosphatidylinositol signalling pathway including IPK2, ITPK2, CAM1-3 and 1 diacylglycerokinase coding gene were induced to be expressed in cold treatment for 1 day. Inhibiting *AtIPK2a* gene by antisense can reportedly promote root growth and pollen germination, *AtIPK2b* can promote shoot branch through the auxin signal-transduction pathway, and the heterologous expression of *AtIPK2b* gene in tobacco can improve the tolerance to abiotic stress [51, 68]. The overexpression and knockout of *OsITPK2* gene in rice showed that ITPK gene negatively regulated drought and salt stress [69]. Calmodulin-coding gene CAM1-3 was induced to be expressed at the T1 phase. It is a calcium-binding protein that exists in almost all eukaryotic cells. It binds with calcium ions to form complexes and then acts on target enzymes to regulate metabolic processes [70]. The calcium signalling pathway and inositol phosphoric acid signalling pathway are induced and expressed in short-term cold treatment. They may play a signalling role in the cold-stress response of rice, and they are ubiquitous amongst different varieties.

Numerous studies have shown that various plant hormones including auxin, JA, and abscisic acid participate in the response to biotic/abiotic stresses [8]. We found that ARFs and auxin-response proteins in the auxin signalling pathway were highly expressed during cold treatment for 1 day. Aux/IAA proteins can act as repressors of early auxin-responsive genes under low auxin solubility. They mediate many physiological and developmental processes in plants. Many DREB/CBF transcription factors have been found in *A. thaliana* to directly regulate the expression of IAA5 and IAA19 genes in response to abiotic stress [71]. Auxin-sensitive Aux/IAA transcription inhibitors and ARF transcription factors synergistically produce complex regulatory networks in plants. By inhibiting ARF transcription factors, auxin-induced gene expression can be regulated [72]. We speculated a special synergistic mechanism between ARFs and auxin-responsive proteins in rice during short-term cold treatment, and regulating auxin-induced related genes can enhance the cold tolerance of rice. Chorismate are intermediates in the biosynthesis of aromatic amino acids in plants and may be involved in salicylic acid biosynthesis [73]. A gene encoding Phospho-2-dehydro-3-deoxyheptonate aldolase was significantly induced at T1 and involved in the first step in the synthesis of chorismate from d-erythritose-4-phosphoric acid and phosphoenolpyruvate. This finding also indirectly proved that chorismate played a potential role in regulating cold tolerance in rice.

Three homologous box leucine zipper proteins (HOX1, HOX18, and HOX32) encoding genes were induced to be expressed in 9311 and DC907 cold treated for 1 day. HOX proteins may be involved in the regulation of seedling growth in plants, and they may be involved in the cold-stress response process in this study. Two mitogen-activated protein kinase (MAPK) coding genes were induced during one day of cold treatment. MAPK is an important signal-transduction module between environmental stimuli and cellular responses. The mutants of MPK4 and MPK6 exhibit reduced cold tolerance [74]. Two Calcium-transporting ATPase enzymes were upregulated significantly during the first day of cold treatment. These magnesium-dependent enzymes catalyse ATP hydrolysis, combine with the transfer of calcium from the cytoplasm to the cytoplasmic reticulum or organelles, and participate in the regulation of cold tolerance [75].

In the process of stress treatment, plant cells lead to the accumulation of various toxic substances. To maintain normal growth in the adverse environment, various pathways may be related to the biosynthesis of detoxifying substances in plants. Seven protein detoxification Coding genes were screened out in 1 day of cold treatment. Four of them were induced in DC907 and 9311. In a low-temperature environment, the removal of toxic substances under early cold stress is one of the molecular bases for maintaining the normal growth of rice seedlings. Notably, during the cold treatment of 9311 and DC907, we screened and identified various genes that may be involved in the regulation of rice cold tolerance. All genes listed above were differently expressed in 9311 and DC907. CASP protein can participate in the regulation of rice growth and development through the dual regulation of Brassinolide and auxin [76]. Three CASP-like proteins (CASP-like proteins) were selected in DC907. Two of them were upregulated and one was downregulated (BLE3), but the expression level was low. This finding suggested that they may participate in the cold-stress response of cold-insensitive rice varieties.

#### Molecular mechanisms of cold stress in two rice species under long-term treatment

In 9311 and DC907 cultivars, numerous genes were differently expressed at the T3 and T3H1 stages compared with CK. These genes may help improve the cold tolerance of DC907 varieties at these stages. The Heat-shock protein (HSP) gene HSP81-1 was upregulated in BT3. This finding indicated that HSP played an important role in increasing the cold-stress tolerance of rice. HSPs are molecular chaperones that can control cell cycle and signal regulation. In rice and wheat, HSP can reportedly impart plants with heat-stress tolerance [77–79]. HSP may also increase cold-stress tolerance in rice. The polar distribution of auxin in plants is related to many developmental processes, including embryogenesis, organogenesis, meristem formation, and polarity establishment. Many articles have focused on auxin's involvement in cold stress, but the molecular mechanism is unknown [80, 81]. Seven WAT1-related proteins were downregulated and one upregulated in BT3, and no significant difference was found in 9311-T3. Two WAT1-related proteins coding genes were downregulated at the AT1 stage and three at the BT1 stage. Walls are thin1 is a plant-specific protein essential for the formation of secondary walls in fibres and plays an important role in auxin transport [82]. It may also prevent SA accumulation in plants [83]. The differential expression of these genes suggests that auxin may play a negative regulatory role in cold stress. At the BT3H1 stage, Germin-like protein 2 coding gene and Oxalate Oxidase 4 coding gene were significantly upregulated. Germin-like proteins are ubiquitous plant proteins that participate in plant growth and development and stress response [84]. Two genes encoding bidirectional sugar transporter SWEET15 and SWEEET2B were upregulated in BT3. The high expression of these genes predicts that the sugar transport and upregulation of sugar metabolism increase plant cold-stress tolerance. E3 ubiquitin-protein ligase may act as a regulator of cell death and defence. Two DEGs existed in BT3, and the overall expression increased. Results showed that it could also help rice play a protective role at low temperature. Eight genes encoding RING-type E3 ubiquitin transferase were upregulated in BT3, suggesting that ubiquitin-mediated programmed cell death could increase cold tolerance in rice. In BT3, we also found four differentially expressed homologous box-leucine zipper protein coding genes (HOX12, HOX18, HOX20, and HOX24). HOX20 had inhibited expression and the other three had induced expression. To adapt to low-temperature environments, plants may need to produce more energy substances and enhance energy-metabolism pathways during cold stress. Numerous genes encoding glycosyltransferases were found in DEGs. In BT3, a large number of genes encoding enzymes such as 3-ketoyl coenzyme A synthase, malic acid synthase, malic acid enzyme, and pyruvate kinase are upregulated in the TCA cycle. Maltose can protect cell membrane under cold stress. In the present study, three genes encoding Beta-amylase were upregulated in BT3. We speculated that the high expression of amylase could protect the cell membrane under cold stress. In abiotic stress, the electron-transport chain is blocked and numerous superoxide ions and acids are produced. Plants remove these toxic substances by expressing large amounts of detoxifying proteins. Nine genes encoding detoxification protein were differently expressed in BT3, most of which were induced, whereas only one gene was upregulated in AT3.

The transcription-factor coding genes BGIOSGA005283 and BGIOSGA019054 of the MYB family are presumed to have very high expression levels in the recovery process of DC907 after 3 days of cold treatment. MYB family transcription factors are ubiquitous in plants. They are also some of the largest transcription families in plants and play an important role in plant metabolism and regulation. Many studies have shown that MYB gene family can respond to abiotic stresses such as drought, salt and low temperature, but no relevant study about their effects on the recovery process of plants after abiotic stress has been conducted. WRKY71 is reportedly involved in biological and abiotic stresses in rice. In this study, WRKY71 was induced in BT3, but not differentially expressed in AT3. We speculated that WRKY71 may be able to regulate gene expression related to cold stress to increase cold tolerance in rice. WRKY transcription factor 58 coding gene was upregulated in BT3, presumably having similar regulatory effect on WKY71.

#### Screening-stage specific module and hub-gene using WGCNA

By WGCNA analysis, we identified a variety-specific module at different stages. At the T1 stage, many genes encoding plant-hormone signal-transduction proteins and transcription factors were highly expressed in this module. We identified four WRKY family transcription factors (WRKY71, 67, 53, and 47) in this module. *TaDREB3* driven by *OsWRKY71* promoter in barley can increase cold resistance and freeze resistance. Two auxin-responsive proteins and two ARFs were related to auxin regulation. The dimer formation of IAA and ARF proteins can alter their ability to regulate early auxin-response gene expression and affect plant growth. Four Homeobox-leucine zipper proteins (HOX1, 11, 16, and 18) were related to GA regulation. Homeobox-leucine zipper transcription factor ATHB6 was identified as a negative regulator of ABA response in *A. thaliana*. Protein TIFY (TI11D, TIFY11A, and TIFY6A) were related to JA regulation. *OsTIFY* gene responds to various abiotic stresses in rice, including drought, salinity, and low temperature, and OsTIFY11a overexpression can increase drought and salt tolerance. The large expression of these genes indicated that they had similar regulation pathways of cold stress under short-term cold stress.

The cyan and dark-grey modules had very different correlations with 9311 and DC907 and had high correlations with DC907. The difference in gene expression between these two modules may have led to the difference in tolerance of the two rice cultivars under one-day cold stress. In the cyan module, Calcium-transporting ATPase gene was identified as a complementary central gene. As a second messenger in plants, Ca^2+^ plays an important role in regulating the expression of downstream genes from environmental stress. SLR1 and Xyloglucan endotransglucosylase/hydrolase genes were identified as candidate central genes in the dark-grey module. SLR1 can reportedly inhibit the GA signalling pathway, leading to plant dwarfing. Thus, inhibiting GA signal transduction can increase rice tolerance to cold stress in a short time.

Stress-regulation genes are highly expressed in the BT3 feature module. They include autophagy-related protein gene, AP2 domain transcription factor coding gene, and HSP gene. Two autophagy-related genes, namely, Atg9 and Atg8A, were identified in the magenta module. The increased expression of autophagic protein in *A. thaliana* could enhance plant tolerance to nitrogen starvation and salt stress. Peptidylprolyl isomerase was identified as a candidate central gene. In *A. thaliana*, Peptidylprolyl isomerase can increase heat tolerance with HSP90.1 and regulate the accumulation of other small HSP chaperones. Hsp81-1 is highly expressed in magenta module and plays an important role in maintaining sugar and starch synthesis under heat stress. The high expression of DREB1B may endow tobacco plants with resistance to high salt, cold, and drought stress. Inserting OsDREB1B gene into tobacco can make plants more tolerant to oxidative and freezing stress than wild type.

In the specific module of BT3H3, we also found a transcription factor gene BGIOSGA029415 encoding the AP2 domain, which transcribes CBF3/DREB1A and ABF3 genes in *A. thaliana* in rice and increases its abiotic-stress tolerance. The identified signal-transduction genes can further enhance our understanding of transcriptional regulation pathways in the process of abiotic stress and recovery after stress and thus provide guidance for the subsequent cultivation of good varieties of rice.

Overall, this study used RNA-seq technology to analyse the cold stress and recovery process in rice. Our findings provided abundant transcriptome data resources for future research on rice crops and new insights for explaining rice cold tolerance and recovery after cold stress from the perspective of histology.

## Methods

### Plant material

In this study, near-isogenic lines DC907 (cold tolerant) and 9311 (not cold tolerant) were used as test materials. They were provided by the *State Key Laboratory for Conservation and Utilisation of Subtropical Agro-bioresources* (Nanning City, China).

Seeds were soaked in 1% carbendazim for 12 h and distilled water for 12 h, and the water was changed every 6 h. The seeds were evenly placed on wet filter paper on top of a petri dish and were germinated at room temperature (25 °C) for 48 h. The uniformly germinated seedlings were transferred into plastic buckets and subjected to hydroponic treatment with Hoagland nutrient solution until the four-leaf stage. For the cold-stress experiment, four-leaf stage rice seedlings were moved into a cold storage treatment and treated with cold stress for 1, 3, and 5 days under simulated low-temperature conditions of 8 ± 0.5 ℃. For the recovery experiment, rice seedlings were removed from cold storage after cold stress and recovered for 1, 3, and 5 days at room temperature. A control experiment was simultaneously conducted. The aerial parts (stems and leaves) of rice were collected at the above time points, immediately quenched with liquid nitrogen, and then stored in a refrigerator at -80 °C for subsequent RNA-seq and q-PCR experiments. The cold resistance of DC907 and 9311 was studied.

### RNA extraction and transcriptome sequencing

Three experimental replicates from 9311 (ACK, AT1, AT1H1, AT3, and AT3H1) and DC907 (BCK, BT1, BT1H1, BT1H3, BT3, BT3H1, and BT3H3) cultivars were used for sequencing. Total RNA was extracted using an RNA Prep Pure Plant Kit (TIANGEN, Beijing, China) following the manufacturer’s protocol. RNA integrity was detected with an Agilent 2100 (Agilent Technologies, CA, USA). Oligo-dT primers and Array Script reverse transcriptase were used to construct the cDNA library. After library construction, initial quantification was performed using Qubit 2.0 to dilute the library to 1 ng/µL (Life Technologies, CA, USA), and then the insert size of the library was detected with Agilent 2100. After the insert size met expectations, the effective concentration of the library was quantified accurately by q-PCR (library effective concentration > 2 nM) to ensure library quality. All samples produced 150 paired terminal-sequence readings on the Illumina platform (Hiseq-PE150).

### Raw data processing, read mapping, and differential gene expression analyses

Raw data in the fastq format were first processed using the NGS QC Toolkit [85]. After removing adapter reads in raw reads, N (i.e., undetermined base information) accounted for more than 10% of reads, and low-quality reads (Qphred ≤ 20 bases accounted for more than 50% of the entire read length) were obtained. Clean reads for subsequent analysis were subsequently obtained. All downstream analyses were based on high-quality clean reads determined by Q30. HISAT2 was used to compare the reads on the rice genome (ftp://ftp.ensemblgenomes.org/pub/plants/release-41/fasta/oryza_indica/dna/) with annotation information (ftp://ftp.ensemblgenomes.org/pub/plants/release-41/gff3/oryza_indica) [33]. Cufflinks were assembled, and a new gene was found by comparing Cuffcomparison with known gene models [86]. The FPKM mapped reads were used to estimate gene expression [87]. The differential expression amongst different samples was determined by DEseq in R package [88, 89]. The P value (padj) < 0.05 of these genes corrected by multiple hypothesis tests was considered to significantly differ in expression.

### Enrichment analysis of GO and Kyoto Encyclopedia of Genes and Genomes (KEGG) and identification of transcription factor

GO enrichment analysis in different genes sets was performed using GOseq in R package [90]. The P value of each GO term was calculated and corrected by multiple-hypothesis test method. The GO terms of corrected p < 0.05 were considered to be significant enrichment. KEGG pathway-enrichment analysis in different genes sets was performed using KOBAS (2.0) [91, 92]. Corrected P value is the corrected value of p after many hypothesis tests. Term of corrected P value < 0.05 is defined as a pathway of significant enrichment. The basic principle of iTAK software for plant transcription-factor prediction is to identify TF by hmmscan using ‘TF’ (transcription factor) family and rules defined in the database. The identification and classification of ‘TF’ are based on Perez-Rodriguez et al. [93].

### QRT-PCR analysis

Isolated total RNA from rice was reversely transcribed into the first-strand cDNA using reverse transcription kit for real-time PCR (TaKaRa). Primers were synthesized by Sangon (Shanghai, China) and were listed in Table S1. The qPCRs were conducted in Step One Real-Time PCR System (Applied Biosystems, USA) using SYBER Premix Ex Taq reagents (TAKARA, Japan) following the program: 95 °C for 30 s, 95 °C for 5 s, and 60 °C for 45 s for 40 cycles. The housekeeping gene β—Actin was used as an internal reference gene, and relative gene expression values were calculated using the 2^−△△Ct^ method based on the data from three biological replicates for each treatment.

### Construction and analysis of co-expression module

We use R-packet WGCNA for co-expression-network analysis [94]. The imported data were the FPKM values of all genes, and all low-expression genes were removed (FPKM < 1 in each treatment; we believed that the gene was not expressed), and the first 75% of the absolute deviation genes were screened, at least MAD was greater than 0.01. The other parameters were selected by default software parameters.

## Supplementary Information

**Additional file 1.**

**Additional file 2.**

**Additional file 3.**

**Additional file 4.**

## Data Availability

The data sets supporting the results of this article are available in the NCBI Gene Expression Omnibus database; under the accession number GSE182563, https://www.ncbi.nlm.nih.gov/geo/query/acc.cgi?acc=GSE182563.

## Abbreviations

ABA: Abscisic Acid
ACK: Control Group of Rice Cultivar 9311
ARF: Auxin Response Factor
AT1: Cold Treatment of Rice Cultivar 9311 for 1 Day
AT1H1: Rice Cultivar 9311 Recovered 1 Day after Cold Treatment for 1 Day
AT3: Cold Treatment of Rice Cultivar 9311 for 3 Day
AT3H1: Rice Cultivar 9311 Recovered 1 Day after Cold Treatment for 3 Day
BCK: Control Group of Rice Cultivar DC907
BT1: Cold Treatment of Rice Cultivar DC907 for 1 Day
BT1H1: Rice Cultivar DC907 Recovered 1 Day after Cold Treatment for 1 Day
BT1H3: Rice Cultivar DC907 Recovered 3 Day after Cold Treatment for 1 Day
BT3: Cold Treatment of Rice Cultivar DC907 for 3 Day
BT3H1: Rice Cultivar DC907 Recovered 1 Day after Cold Treatment for 3 Day
BT3H3: Rice Cultivar DC907 Recovered 3 Day after Cold Treatment for 3 Day
CDPK: Calcium-dependent Protein Kinase
DEG(s): Differentially Expressed Gene(s)
FPKM: Fragments Per Kilobase of Transcript Length Per Million Mapped Reads
GO: Gene Ontology
GSTU: Glutathione S-transferase
H1: Normal Temperature Recovery Treatment for 1 Day
H3: Normal Temperature Recovery Treatment for 3 Day
KEGG: Kyoto Encyclopedia of Genes and Genomes
Kme: Module Eigengene Connectivity
MAPK: Mitogen-activated Protein Kinase
NCED: 9-Cis-epoxycarotenoid Dioxygenase
QTLs: Quantitative trait locus
ROS: Reactive Oxygen Species
SAPK2: Serine/threonine-protein Kinase2
T1: Cold Stress Treatment for 1 Day
T3: Cold Stress Treatment for 3 Day
TF(s): Transcription Factor(s)
WGCNA: Weighted gene co-expression network analysis
