# Supplementary material for: Gene-coexpression network analysis identifies specific modules and hub genes related to cold stress in rice
Source: BMC Genomics. 2022 Apr 1;23:251. doi: 10.1186/s12864-022-08438-3 (PMC8974213; doi:10.1186/s12864-022-08438-3)
Supplement: Supplementary file 1 — Additional file 1. [file 12864_2022_8438_MOESM1_ESM.docx]

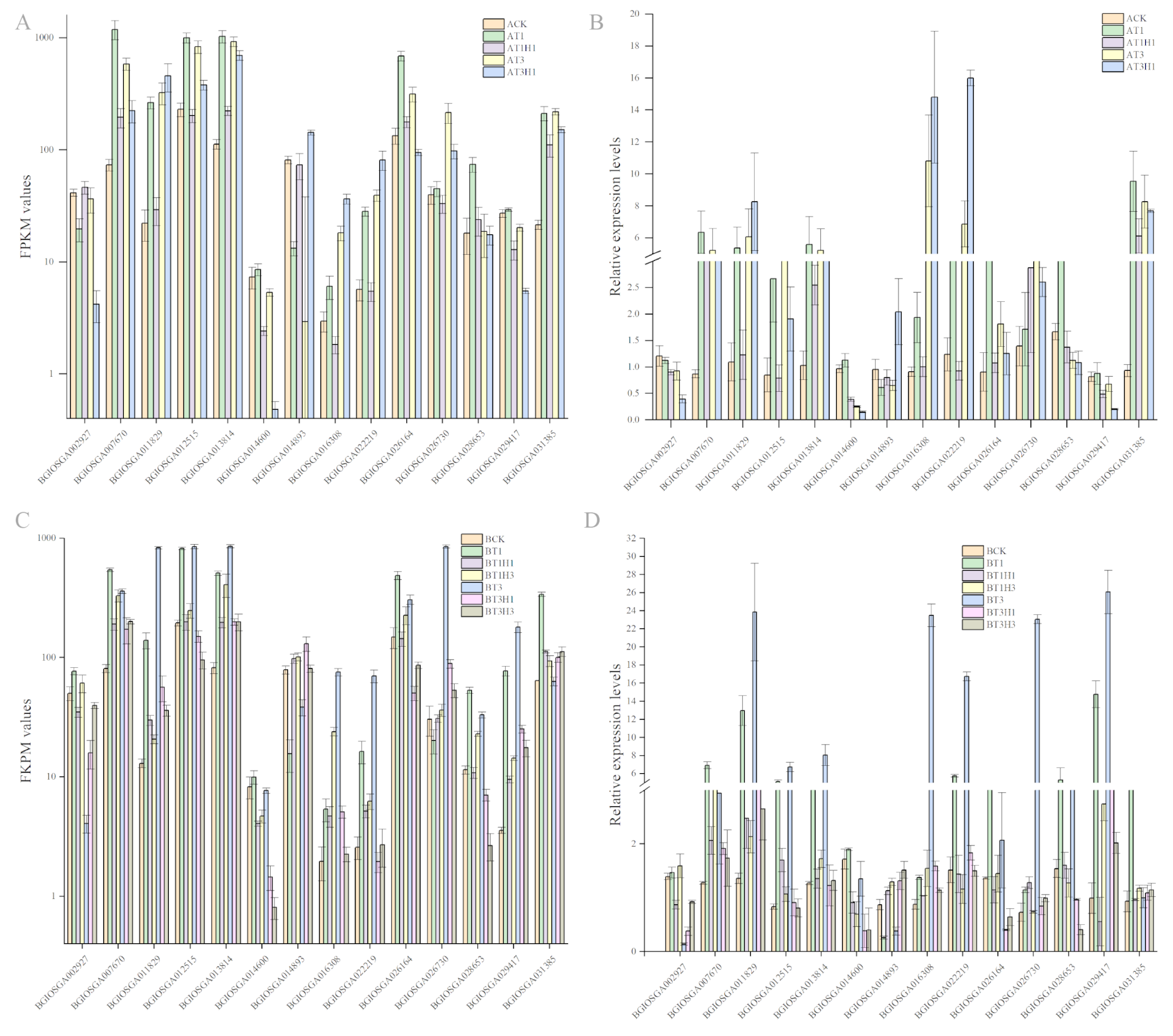


**Fig. S1** The FKPM values and relative levels of 14 genes. The FKPM values of 14 genes in 9311 (A) and DC907 (C) treated with cold treatments by sequencing. The relative expression levels of 14 genes in 9311 (B) and DC907 (D) treated with cold treatments by qRT-PCR.
