# Supplementary material for: Gene-coexpression network analysis identifies specific modules and hub genes related to cold stress in rice
Source: BMC Genomics. 2022 Apr 1;23:251. doi: 10.1186/s12864-022-08438-3 (PMC8974213; doi:10.1186/s12864-022-08438-3)
Supplement: Supplementary file 2 — Additional file 2. [file 12864_2022_8438_MOESM2_ESM.docx]

**Table S1** Primers used in this study.

| Genes ID | Primers sequences (5′ - 3′) | |
| --- | --- | --- |
|  | Forward | Reverse |
| BGIOSGA002927 | ACCTTGCCTCGTCGTCGTCATC | TTGCTCGCCTTGTCGCCGTT |
| BGIOSGA007670 | GGCAACAACCAGTCGTCCACCT | CCCATCTTTCACCACCAGGCTGAG |
| BGIOSGA011829 | GGTACTCGCAGATGCTCCTGGAAC | CGTCGTGGTGCCTCGTGTTGTA |
| BGIOSGA012515 | ACGCAAGATGAAGGACACCGACTC | GCTTCTCGCCGAGGTTGGTCAT |
| BGIOSGA013814 | GATTGGGTGCTGTGTCGGCTGTA | ATGTCGGACGCCTCCTCCTTCA |
| BGIOSGA014600 | AGACGGAGGTGGACTGCGAGTT | TTCATGTAGAGGTGCGGCGAGAC |
| BGIOSGA014893 | TCTAGGGAGGCGAGGATATGGAAGG | CTGGATGAGGAGGTGGGAGTAGGAG |
| BGIOSGA016308 | CGCTCCGATGACGCTCTTCTACAA | TTAGTTGCCTCGGTTGCCATCCTC |
| BGIOSGA022219 | CCCGCCATGATGATGCAGTACCA | GGTCGAAGTCCATGTTGCCGTAGT |
| BGIOSGA026164 | GATGTGCGGACCAAGGAGCTGTT | CAATGTTCGGATGCCTCAGTGACCT |
| BGIOSGA026730 | ACTGGCTGCTGGTGCTGATGTG | TGCTCGTCATCGTTGTGCTTGGT |
| BGIOSGA028653 | GCATCGGCATCCTCGTGTCCAA | CCGCAGCAAGAACTCCATCACCT |
| BGIOSGA029417 | AACAAGGACGTGTTGCCGGTGG | TGCGCCAAGCTCGCGTAGTA |
| BGIOSGA031385 | GCGACACATTCTGCGGCTACCT | CGAAGGATACCTCAGCTCACCACAA |
